# Stratifying Type 2 Diabetes Cases by BMI Identifies Genetic Risk Variants in *LAMA1* and Enrichment for Risk Variants in Lean Compared to Obese Cases

**DOI:** 10.1371/journal.pgen.1002741

**Published:** 2012-05-31

**Authors:** John R. B. Perry, Benjamin F. Voight, Loïc Yengo, Najaf Amin, Josée Dupuis, Martha Ganser, Harald Grallert, Pau Navarro, Man Li, Lu Qi, Valgerdur Steinthorsdottir, Robert A. Scott, Peter Almgren, Dan E. Arking, Yurii Aulchenko, Beverley Balkau, Rafn Benediktsson, Richard N. Bergman, Eric Boerwinkle, Lori Bonnycastle, Noël P. Burtt, Harry Campbell, Guillaume Charpentier, Francis S. Collins, Christian Gieger, Todd Green, Samy Hadjadj, Andrew T. Hattersley, Christian Herder, Albert Hofman, Andrew D. Johnson, Anna Kottgen, Peter Kraft, Yann Labrune, Claudia Langenberg, Alisa K. Manning, Karen L. Mohlke, Andrew P. Morris, Ben Oostra, James Pankow, Ann-Kristin Petersen, Peter P. Pramstaller, Inga Prokopenko, Wolfgang Rathmann, William Rayner, Michael Roden, Igor Rudan, Denis Rybin, Laura J. Scott, Gunnar Sigurdsson, Rob Sladek, Gudmar Thorleifsson, Unnur Thorsteinsdottir, Jaakko Tuomilehto, Andre G. Uitterlinden, Sidonie Vivequin, Michael N. Weedon, Alan F. Wright, MAGIC, DIAGRAM Consortium, GIANT Consortium, Frank B. Hu, Thomas Illig, Linda Kao, James B. Meigs, James F. Wilson, Kari Stefansson, Cornelia van Duijn, David Altschuler, Andrew D. Morris, Michael Boehnke, Mark I. McCarthy, Philippe Froguel, Colin N. A. Palmer, Nicholas J. Wareham, Leif Groop, Timothy M. Frayling, Stéphane Cauchi

**Affiliations:** 1 Genetics of Complex Traits, Peninsula Medical School, University of Exeter, Exeter, United Kingdom; 2 Wellcome Trust Centre for Human Genetics, University of Oxford, Oxford, United Kingdom; 3 Department of Twin Research and Genetic Epidemiology, King's College London, London, United Kingdom; 4 Broad Institute of Harvard and Massachusetts Institute of Technology, Cambridge, Massachusetts, United States of America; 5 CNRS UMR 8199, Genomics of Metabolic Diseases, Lille, France; 6 Department of Epidemiology, Erasmus MC, Rotterdam, The Netherlands; 7 Department of Biostatistics, Boston University School of Public Health, Boston, Massachusetts, United States of America; 8 National Heart, Lung, and Blood Institute's Framingham Heart Study, Framingham, Massachusetts, United States of America; 9 Department of Biostatistics and Center for Statistical Genetics, University of Michigan, Ann Arbor, Michigan, United States of America; 10 Research Unit of Molecular Epidemiology, Helmholtz Zentrum Muenchen, Neuherberg, Germany; 11 MRC Human Genetics Unit, Medical Research Council Institute of Genetics and Molecular Medicine, University of Edinburgh, Edinburgh, United Kingdom; 12 Johns Hopkins Bloomberg School of Public Health and Epidemiology, Baltimore, Maryland, United States of America; 13 Departments of Nutrition and Epidemiology, Harvard School of Public Health, Boston, Massachusetts, United States of America; 14 deCODE Genetics, Reykjavik, Iceland; 15 MRC Epidemiology Unit, Medical Research Council, Cambridge, United Kingdom; 16 Diabetes and Endocrinology Research Unit, Department of Clinical Sciences, Lund University, Malmoe, Sweden; 17 McKusick-Nathans Institute of Genetic Medicine, Johns Hopkins University School of Medicine, Baltimore, Maryland, United States of America; 18 INSERM CESP U1018, Villejuif, France; 19 Landspitali University Hospital, Reykjavik, Iceland; 20 Icelandic Heart Association, Kopavogur, Iceland; 21 Diabetes and Obesity Research Institute, Cedars-Sinai Medical Center, Los Angeles, California, United States of America; 22 University of Texas Health Science Center at Houston, Human Genetics Center, Houston, Texas, United States of America; 23 National Human Genome Research Institute, National Institutes of Health, Bethesda, Maryland, United States of America; 24 Centre for Population Health Sciences, University of Edinburgh, Teviot Place, Edinburgh, United Kingdom; 25 Corbeil-Essonnes hospital, Department of Endocrinology-Diabetology, Corbeil-Essonnes, France; 26 Institute of Genetic Epidemiology, Helmholtz Zentrum Muenchen, Neuherberg, Germany; 27 CHU Poitiers, Department of Endocrinology-Diabetology, CIC INSERM 0801, INSERM U927, University of Medical and Pharmaceutical Sciences, Poitiers, France; 28 Institute for Clinical Diabetology, German Diabetes Center, Leibniz Center for Diabetes Research, Heinrich Heine University Düsseldorf, Düsseldorf, Germany; 29 Freiburg University Clinic, Renal Division, Freiburg, Germany; 30 Boston University School of Public Health, Boston, Massachusetts, United States of America; 31 Department of Genetics, University of North Carolina, Chapel Hill, North Carolina, United States of America; 32 Erasmus University Medical School, Rotterdam, The Netherlands; 33 School of Public Health, Division of Epidemiology and Community Health, University of Minnesota, Minneapolis, Minnesota, United States of America; 34 Center for Biomedicine, European Academy Bozen/Bolzano (EURAC), Bolzano, Italy (Affiliated Institute of the University of Lübeck, Lübeck, Germany); 35 Department of Neurology, General Central Hospital, Bolzano, Italy; 36 Department of Neurology, University of Lübeck, Lübeck, Germany; 37 Oxford Centre for Diabetes, Endocrinology, and Metabolism, University of Oxford, Oxford, United Kingdom; 38 Institute of Biometrics and Epidemiology, German Diabetes Center, Leibniz Center for Diabetes Research, Heinrich Heine University Düsseldorf, Düsseldorf, Germany; 39 Department of Metabolic Diseases, University Hospital Düsseldorf, Düsseldorf, Germany; 40 Centre for Population Health Sciences, University of Edinburgh, Edinburgh, United Kingdom; 41 Boston University Data Coordinating Center, Boston, Massachusetts, United States of America; 42 Department of Human Genetics, Faculty of Medicine, McGill University, Montreal, Canada; 43 Faculty of Medicine, University of Iceland, Reykjavík, Iceland; 44 Diabetes Prevention Unit, National Institute for Health and Welfare, Helsinki, Finland; 45 South Ostrobothnia Central Hospital, Seinäjoki, Finland; 46 Red RECAVA Grupo RD06/0014/0015, Hospital Universitario La Paz, Madrid, Spain; 47 Centre for Vascular Prevention, Danube-University Krems, Krems, Austria; 48 Department of Internal Medicine, Erasmus MC, Rotterdam, The Netherlands; 49 Hannover Unified Biobank, Hannover Medical School, Hannover, Germany; 50 General Medicine Division, Massachusetts General Hospital and Department of Medicine, Harvard Medical School, Boston, Massachusetts, United States of America; 51 Biomedical Research Institute, Ninewells Hospital and Medical School, University of Dundee, Dundee, United Kingdom; 52 Oxford NIHR Biomedical Research Centre, Churchill Hospital, Oxford, United Kingdom; 53 Department of Genomics of Common Diseases, Hammersmith Hospital, Imperial College London, London, United Kingdom; Georgia Institute of Technology, United States of America

## Abstract

Common diseases such as type 2 diabetes are phenotypically heterogeneous. Obesity is a major risk factor for type 2 diabetes, but patients vary appreciably in body mass index. We hypothesized that the genetic predisposition to the disease may be different in lean (BMI<25 Kg/m^2^) compared to obese cases (BMI≥30 Kg/m^2^). We performed two case-control genome-wide studies using two accepted cut-offs for defining individuals as overweight or obese. We used 2,112 lean type 2 diabetes cases (BMI<25 kg/m^2^) or 4,123 obese cases (BMI≥30 kg/m^2^), and 54,412 un-stratified controls. Replication was performed in 2,881 lean cases or 8,702 obese cases, and 18,957 un-stratified controls. To assess the effects of known signals, we tested the individual and combined effects of SNPs representing 36 type 2 diabetes loci. After combining data from discovery and replication datasets, we identified two signals not previously reported in Europeans. A variant (rs8090011) in the *LAMA1* gene was associated with type 2 diabetes in lean cases (P = 8.4×10^−9^, OR = 1.13 [95% CI 1.09–1.18]), and this association was stronger than that in obese cases (P = 0.04, OR = 1.03 [95% CI 1.00–1.06]). A variant in *HMG20A*—previously identified in South Asians but not Europeans—was associated with type 2 diabetes in obese cases (P = 1.3×10^−8^, OR = 1.11 [95% CI 1.07–1.15]), although this association was not significantly stronger than that in lean cases (P = 0.02, OR = 1.09 [95% CI 1.02–1.17]). For 36 known type 2 diabetes loci, 29 had a larger odds ratio in the lean compared to obese (binomial P = 0.0002). In the lean analysis, we observed a weighted per-risk allele OR = 1.13 [95% CI 1.10–1.17], P = 3.2×10^−14^. This was larger than the same model fitted in the obese analysis where the OR = 1.06 [95% CI 1.05–1.08], P = 2.2×10^−16^. This study provides evidence that stratification of type 2 diabetes cases by BMI may help identify additional risk variants and that lean cases may have a stronger genetic predisposition to type 2 diabetes.

## Author Summary

Individuals with Type 2 diabetes (T2D) can present with variable clinical characteristics. It is well known that obesity is a major risk factor for type 2 diabetes, yet patients can vary considerably—there are many lean diabetes patients and many overweight people without diabetes. We hypothesized that the genetic predisposition to the disease may be different in lean (BMI<25 Kg/m^2^) compared to obese cases (BMI≥30 Kg/m^2^). Specifically, as lean T2D patients had lower risk than obese patients, they must have been more genetically susceptible. Using genetic data from multiple genome-wide association studies, we tested genetic markers across the genome in 2,112 lean type 2 diabetes cases (BMI<25 kg/m^2^), 4,123 obese cases (BMI≥30 kg/m^2^), and 54,412 healthy controls. We confirmed our results in an additional 2,881 lean cases, 8,702 obese cases, and 18,957 healthy controls. Using these data we found differences in genetic enrichment between lean and obese cases, supporting our original hypothesis. We also searched for genetic variants that may be risk factors only in lean or obese patients and found two novel gene regions not previously reported in European individuals. These findings may influence future study design for type 2 diabetes and provide further insight into the biology of the disease.

## Introduction

Common diseases such as type 2 diabetes are highly phenotypically heterogeneous. Few studies have performed genome wide association studies in subsets of patients defined by more stringent phenotypic characteristics. It is possible that reducing the heterogeneity of disease cases may increase power to detect associations over and above the loss of power resulting from reduced numbers. To address these questions we hypothesized that the genetic predisposition to Type 2 diabetes may be different in two strata of cases defined by well-accepted cut-offs for body mass index, the strongest known risk factor for type 2 diabetes.

Genome-wide association (GWA) studies have identified ∼50 independent loci robustly associated with type 2 diabetes [1], [2], [3], [4], [5], [6], [7]. These studies have highlighted new candidate pathways involved in the disease [8], [9], identified overlap with monogenic forms of the disease [1], and provided genetic links with correlated phenotypes [10], [11].

The GWA studies of type 2 diabetes have not so far provided a greatly improved understanding of the clinical heterogeneity of the disease. Type 2 diabetes cases vary appreciably in their clinical characteristics, particularly age of diagnosis and body mass index (BMI). There is also a group of patients who may present with evidence of an autoimmune component to their diabetes, but who are not insulin dependent [12]. In contrast, the identification of the genetic component to monogenic forms of diabetes has often explained the clinical heterogeneity observed [13].

Previous studies have provided some evidence of genetic heterogeneity between non-obese and obese type 2 diabetic cases [14], [15], [16], [17]. For example, the variant with the strongest effect on type 2 diabetes risk, in *TCF7L2*, has a stronger effect in non-obese cases (odds ratio = 1.53 [0.37–1.71] compared to obese cases (OR = 1.21 [1.09–1.35]) [14]. The effect of *FTO* variation on type 2 diabetes risk depends on how cases and controls are ascertained by BMI status, but this was expected given *FTO*'s known primary effect on BMI. In the most recent GWA studies of type 2 diabetes [1], risk variants tended to have stronger effects in non-obese compared to obese individuals – of 30 loci examined, 23 showed stronger associations in non-obese compared to obese individuals.

We designed the present study in an attempt to understand better the genetic heterogeneity of type 2 diabetes. Type 2 diabetes GWA studies tend to be enriched with cases with stronger family histories and lower average BMIs compared to community based studies. Nevertheless, there is a wide spectrum of BMI amongst type 2 diabetes cases used in GWA studies, with more cases being obese than lean. In this study we tested the hypothesis that we would identify new genetic variants by limiting the clinical heterogeneity of type 2 diabetes. By stratifying cases by their BMI status and performing separate GWA studies for each strata of BMI we identified two signals of association not previously reported in the largest GWA studies in Europeans [1], although one signal has been identified in a South Asian study [7]. In addition we confirmed with additional data that the majority of known type 2 diabetes genetic associations have stronger effects in lean type 2 diabetic cases compared to obese cases.

## Methods

Descriptions of all cases are available in Table 1, and combined with control details in Tables S1 and S2. Our study was designed to limit the clinical heterogeneity of type 2 diabetes by stratification on BMI, whilst also using the largest sample sizes available:

**Table 1:** Patient characteristics for discovery and replication type 2 diabetes case samples.

	Lean Patients				Obese Patients			
Study	N	M/F	Age Diag	BMI mean SD	N	M/F	Age Diag	BMI mean SD
**GWA studies discovery**								
DGI	225	106/119	59.47 (10.57)	22.93 (1.51)	303	143/160	56.49 (9.90)	33.10 (2.52)
WTCCC	257	160/97	n/a	23.00 (1.54)	1,011	533/478	n/a	35.63 (4.98)
FUSION	123	78/45	53.67 (9.73)	23.22 (1.61)	529	265/264	53.62 (8.72)	34.00 (3.37)
deCODE	214	117/97	54.60 (14.90)	23.20 (1.78)	625	346/279	54.10 (11.20)	34.69 (4.38)
KORA	36	21/15	57.48 (11.92)	23.47 (1.33)	219	115/104	56.77 (9.82)	34.64 (4.07)
DGDG	185	99/86	44.30 (9.13)	22.72 (1.81)	-	-	-	-
Rotterdam	301	144/157	n/a	22.87 (1.58)	247	62/185	n/a	33.05 (3.02)
Eurospan-MICROS	-	-	-	-	22	15/7	n/a	34.30 (3.65)
Eurospan-Orcades	-	-	-	-	21	14/7	n/a	35.25 (4.52)
Eurospan-ERF	-	-	-	-	25	14/11	n/a	34.98 (3.80)
Eurospan-Vis	-	-	-	-	38	25/13	n/a	32.83 (2.74)
FHS	93	47/46	58	23.07	331	181/150	56.00	36.27
ARIC	111	52/59	47.90 (12.00)	23.06 (1.50)	358	174/178	51.40 (9.35)	34.95 (4.27)
NHS	567	0/100	57.68 (13.37)	22.67 (1.67)	394	0/100	56.01 (10.10)	33.88 (3.77)
*Total*	*2,112*		*54.14 (11.66)*	*23.02 (1.59)*	*4,123*		*54.91 (9.84)*	*34.43 (3.76)*
**Replication**								
GoDarts	263	151/112	56.83 (8.95)	22.97 (1.77)	1,735	950/785	54.8 (8.96)	35.94 (5.33)
DGDG	1,161	680/530	48.00 (10.02)	22.53 (1.51)	2,972	1,599/1,504	49.00 (11.12)	34.43 (4.36)
Malmo CC	477	291/186	59.2 (11.6)	22.9 (1.9)	1080	583/497	55.8 (10.8)	34.70 (4.30)
ADDITION-Ely	39	27/12	66.59 (5.20)	23.31 (1.57)	586	346/240	60.67 (7.65)	35.69 (4.78)
EPIC-NDCCS	941	544/397	63.35 (12.29)	22.96 (1.76)	2,329	1,208/1,121	58.01 (11.25)	35.04 (4.81)
*Total*	*2,881*		*58.79 (9.61)*	*22.93 (1.7)*	*8,702*		*55.66 (9.96)*	*35.16 (4.72)*

Eurospan represents a single cohort in the main text, however is split into its component studies in this table. There were too few lean cases in the Eurospan studies to include in meta-analysis. - = individuals not used. n/a = individuals used in analyses but data not available.

### Study design—choice of strata

To test the hypothesis that we would identify new variants associated with type 2 diabetes in different BMI strata, we used the following study design. We used two separate strata of type 2 diabetes cases defined by the two arbitrary, but well established, cut-offs for classifying people as overweight or obese. The first stratum consisted of non-overweight cases, here defined as “lean” (BMI<25 kg/m^2^). The second strata consisted of obese cases (BMI≥30 kg/m^2^). For each stratum we used all controls, not selected on BMI to increase statistical power and provide a more robust estimate of the population allele frequency. We did not correct for BMI as BMI was not available in all controls. To check whether or not associations were being driven primarily by effects on BMI we assessed novel variants in an existing GWA studies of BMI using 123,865 individuals from the GIANT consortium [18]. Finally, we performed sensitivity analyses, confirming our findings by stratifying controls by BMI as well as cases.

### Study design—choice of studies

We chose to include the largest set of studies available. These studies differed in the proportion of total cases defined as lean (8.4–30.4%), the proportion of total cases defined as obese (21.2–77.8%, plus one GWA study, DGDG, that only selected non-obese cases). Some studies were specifically designed as case control studies and some as case-cohort studies, and we note that the extent of phenotyping performed to exclude autoimmune processes was different across studies, ranging from not requiring insulin treatment in the first year of diagnosis and GAD autoantibody negative, to general practitioner diagnosis of type 2 diabetes.

Descriptions of the participating studies are available in the most recent DIAGRAM manuscript [1], with summary statistics also presented in Table 1 and in Tables S1 and S2. The two discovery GWA study meta-analyses comprised 2112 lean type 2 diabetes cases or 4123 obese type 2 diabetes cases, compared against up to 54,412 controls. For a subset of SNPs available on the Metabochip (a custom Illumina iSelect SNP array that included the SNPs identified by GWA studies for several diseases and traits including type 2 diabetes loci) we included data from an additional 263 lean type 2 diabetes cases, 1735 obese type 2 diabetes cases, and 3691 controls from the GoDARTs study [19].

### GWA study methodology

With the exception of the BMI-stratification of cases, the meta-analyses, individual study quality control, and analytical methods were the same as those recently reported [1]. A genomic control inflation factor was calculated for each study for each analysis, and their test statistics were adjusted accordingly. Inverse-variance fixed effect meta-analyses were performed on imputed SNP datasets, testing for an additive genetic effect. All single point effect estimates are given with their [95% confidence intervals (CI)]. Only autosomal SNPs with imputation quality scores >0.5 and a minor allele frequency >1% were included from each study. A SNP was excluded from the meta-analysed dataset if it was present in less than half of the studies. Given the use of two strata, we used a p-value threshold of 2.5×10^−8^ as the criterion for genome-wide significance.

### Follow-up studies—replication of novel associations in lean and obese GWA studies

An additional 4 studies, totalling 2881 lean cases, 8702 obese cases, and 18957 controls were available for *de novo* genotyping of SNPs (Table S2). For the DGDG replication, all polymorphisms were genotyped using the KASPar system (KBiosciences). For Malmo CC, ADDITION-Ely, and Norfolk Diabetes Case Control Study (NDCCS), Taqman assay genotyping was performed. For all four studies genotyping success rate was >95%, the genotyping error rate was 0% based on re-genotyping of 384 individuals, and all SNPs were in Hardy-Weinberg equilibrium (*P*>0.05). We re-performed the inverse-variance weighted meta-analysis for the replication SNPs using data from all the discovery and replication datasets.

### Association of variants with BMI

To test whether or not type 2 diabetes associations could be primarily driven by effects on BMI, we assessed the association of novel SNPs with BMI using data from the GIANT consortium consisting of 123,865 individuals.

### Association of variants in case-only analyses

There are two possible reasons why a variant may be associated with type 2 diabetes in a stratified sample compared to using all data. First, the variant may have a genuinely larger effect in that stratum compared to the overall sample. Second, chance will influence which SNPs are most strongly associated in different subsets of data. To distinguish between these two possibilities we performed a case only analysis in which we tested whether variants associated with lean or obese type 2 diabetes were also associated with BMI within type 2 diabetes cases. We analysed BMI as a quantitative trait in cases from the GWA studies and meta-analysed the summary statistics. If a variant is genuinely associated with type 2 diabetes with stronger effects in the lean stratum, for example, we would expect the risk allele to be associated with lower BMI within cases. This phenomenon was previously reported for the variant in *TCF7L2* [14].

### Continuous glycaemic measures

SNP association statistics on glyacemic traits in healthy individuals were provided by the Meta-Analyses of Glucose and Insulin-related traits Consortium (MAGIC). Phenotypes available were fasting insulin (N = 38,238, fasting glucose (N = 46,186), beta-cell function (HOMA-B, N = 36,466), insulin resistance (HOMA-IR, N = 37,037), HbA_1C_ (N = 46,368) and 2 hour glucose (N = 15,234) after an oral glucose challenge. All traits are naturally log transformed, besides fasting glucose, 2 hour glucose and HbA1c. The studies and methodology for these GWA study data are described in their recent publications [2], [20], [21] and available online at www.magicinvestigators.org. We also had access to data from joint meta-analyses of SNP and SNPxBMI interaction on fasting glucose (N = 58,074), insulin (N = 51,570), and 2-hr glucose (N = 15,141), also provided by MAGIC (Manning *et al*, in press).

### eQTL assessment

Identified SNPs were searched against a collected database of expression SNP (eQTL) results including a range of tissues [22], [23], [24], [25], [26], [27], [28], [29], [30], [31], [32], [33], [34], [35], [36], [37], [38].

### Testing the role of known SNPs in lean and obese individuals

In addition to identifying new loci, we tested the impact of BMI stratification on SNPs previously identified as associated with type 2 diabetes. We calculated the individual SNP association statistics using the lean and obese meta-analyses described above.

To assess the effects of combining information from all known type 2 diabetes SNPs, we next used a single study, the GoDARTs [19] study, independent from the discovery GWA studies. In GoDARTs there were a total of 263 lean type 2 diabetes cases, 1735 obese type 2 diabetes cases, and 3691 controls. Known SNPs (N = 36 on the metabochip) were defined as those reaching genome-wide significance in studies using samples of European descent (excluding *FTO* due to primary effect on BMI, and *DUSP9* not present on the chip) [1], [2], [3]. We also combined the 36 SNPs into a single allele count model. This analysis consisted of a logistic regression model comparing the count of an individual's type 2 diabetes risk alleles, against case-control status. Each risk allele count was weighted by the point estimate effect size of that SNP from the DIAGRAM meta-analysis [1]. We repeated this analysis using stratified controls (BMI<25 kg/m^2^ versus lean cases and BMI≥30 kg/m^2^ versus obese cases) instead of all controls. Finally, individuals were binned into quintiles based on their weighted allele score and per-quintile odds ratios calculated.

## Results

### Genome-wide association in lean type 2 diabetic individuals

Three independent association signals reached *P*<2.5×10^−8^ in the lean case genome wide meta-analysis (Table 2). Two represented previously reported loci - *TCF7L2* (OR = 1.58 [1.47–1.68], *P* = 2×10^−40^) and *CDKAL1* (OR = 1.26 [1.17–1.35], *P* = 7×10^−10^). One novel locus reached genome-wide significance, lead SNP positioned ∼25 kb from the *HLA-DQA2* gene (OR = 1.3 [1.19–1.42], *P* = 1×10^−8^). Three further independent signals reached *P*<5×10^−7^, two of which were previously identified (SNPs in or near *ADCY5*, OR = 1.25 [1.15–1.35] *P* = 6×10^−8^, and *SLC30A8*, OR = 1.23 [1.15–1.33] *P* = 4×10^−8^) and one of which was novel (SNPs in *LAMA1*, OR = 1.22 [1.12–1.30] *P* = 1×10^−7^). Rs numbers are given in Table 2.

**Table 2:** Highest-ranked independent signals in the lean and obese case GWA studies.

				Discovery						Replication			Combined		
Lean Analysis				Lean			Obese			Lean					
SNP	Nr Gene	Risk Allele	RAF	OR	P-Value	N Case/ctrl	OR	P-Value	N Case/ctrl	OR	P-value	N	P-value	OR	N
rs7903146	*TCF7L2*	t	0.29	1.58 [1.47–1.68]	2.00E−40	2375/55249	1.26 [1.2–1.32]	4.40E−21	5858/58103	n/a	n/a	n/a	n/a	n/a	n/a
rs7766070	*CDKAL1*	a	0.27	1.26 [1.17–1.35]	7.30E−10	2112/51558	1.21 [1.14–1.28]	5.80E−11	5858/58103	n/a	n/a	n/a	n/a	n/a	n/a
rs3916765	*HLA-DQA2*	a	0.12	1.3 [1.19–1.42]	1.20E−08	2375/55249	1.07 [1.00–1.15]	0.04	5858/58103	0.96 [0.81–1.13]	0.62	1161/3960	1.00E−06	1.21 [1.12–1.31]	3536/59209
rs3802177	*SLC30A8*	g	0.68	1.23 [1.15–1.33]	3.80E−08	2082/50879	1.12 [1.06–1.19]	5.03E−05	5858/58103	n/a	n/a	n/a	n/a	n/a	n/a
rs11708067	*ADCY5*	a	0.78	1.25 [1.15–1.35]	5.70E−08	2375/55249	1.07 [1.01–1.14]	0.01	5858/58103	n/a	n/a	n/a	n/a	n/a	n/a
rs8090011	*LAMA1*	g	0.38	1.22 [1.12–1.3]	1.00E−07	2112/51558	1.02 [0.96–1.08]	0.5	5858/58103	1.09 [1.03–1.15]	0.003	2881/18957	8.40E−09	1.13 [1.09–1.18]	4993/70515

Obese Analysis				Obese			Lean			Obese					
SNP	Nr Gene	Risk Allele	RAF	OR	P-Value	N Case/ctrl	OR	P-Value	N Case/ctrl	OR	P-value	N	P-value	OR	N
rs7903146	*TCF7L2*	t	0.29	1.26 [1.2–1.32]	4.40E−21	5858/58103	1.58 [1.47–1.68]	2.00E−40	2375/55249	n/a	n/a	n/a	n/a	n/a	n/a
rs9939609	*FTO*	a	0.4	1.25 [1.19–1.3]	1.10E−20	5858/58103	0.95[0.89–1.02]	0.16	2375/55249	n/a	n/a	n/a	n/a	n/a	n/a
rs7766070	*CDKAL1*	a	0.27	1.21 [1.14–1.28]	5.80E−11	4123/54412	1.26 [1.17–1.35]	7.30E−10	2375/55249	n/a	n/a	n/a	n/a	n/a	n/a
rs5015480	*HHEX*	c	0.57	1.18 [1.11–1.23]	1.90E−09	4123/54412	1.17[1.09–1.25]	6.15E−06	2375/55249	n/a	n/a	n/a	n/a	n/a	n/a
rs4402960	*IG2BP2*	t	0.31	1.15 [1.1–1.21]	2.50E−09	5858/58103	1.16[1.08–1.24]	1.53E−05	2375/55249	n/a	n/a	n/a	n/a	n/a	n/a
rs6567160	*MC4R*	c	0.23	1.15 [1.09–1.2]	1.80E−07	5858/58103	1.07[0.99–1.15]	0.07	2375/55249	n/a	n/a	n/a	n/a	n/a	n/a
rs7178572	*HMG20A*	g	0.7	1.14 [1.09–1.19]	2.10E−07	5858/58103	1.09[1.02–1.16]	0.01	2375/55249	1.09 [1–1.16]	0.04	2972/3960	1.30E−08	1.11 [1.07–1.15]	8583/62063
rs16896390	*ANKS1A*	c	0.91	1.3 [1.18–1.43]	4.70E−07	4123/54412	1.14[1.00–1.29]	0.04	2375/55249	0.97 [0.89–1.06]	0.55	4707/7651	0.002	1.11 [1.04–1.19]	8583/62063

SNPs mapped to ‘+’ strand, genome build 36. Independence based on hapmap r^2^<0.05. Study directions show directional consistency of effect size estimates within the individual cohorts meta-analysed.

### Genome-wide association in obese type 2 diabetic individuals

In the obese case genome wide meta-analysis, five signals reached genome-wide significance (Table 2), all in or near known loci *TCF7L2*, *FTO*, *CDKAL1*, *HHEX*, and *IGF2BP2*. A further three signals reached *P*<5×10^−7^; SNPs in or near the *MC4R* gene (previously associated with BMI), and two other signals; in *HMG20A* (previously reported in South Asians -OR = 1.14 [1.09–1.19] *P* = 2×10^−7^) and in *ANKS1A* (OR = 1.3 [1.18–1.43] *P* = 5×10^−7^).

### Follow-up of putative novel signals

**Figure 1:**
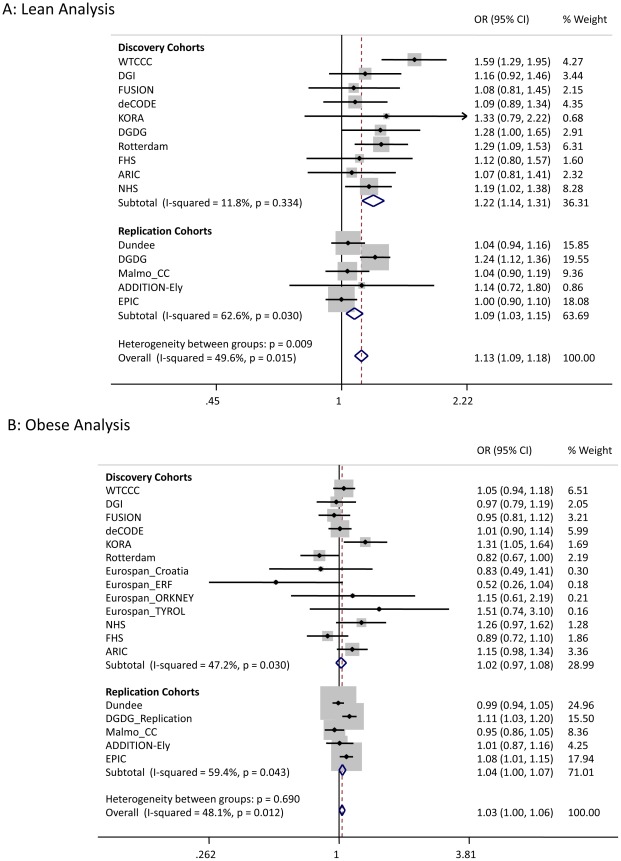
Test statistics for LAMA1 association in lean and obese cases versus all controls.

**Figure 2:**
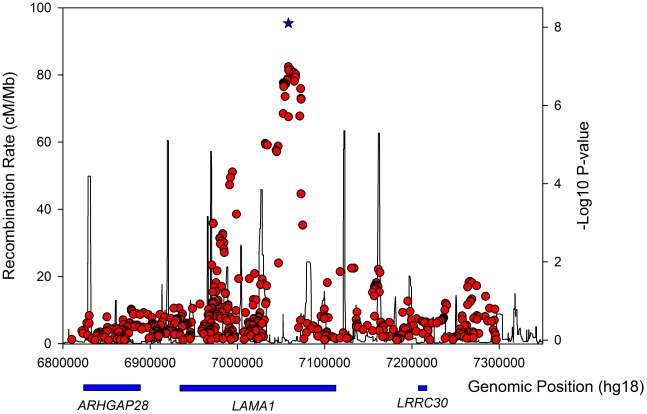
Regional association plot for the *LAMA1* gene in lean type 2 diabetes samples.

**Figure 3:**
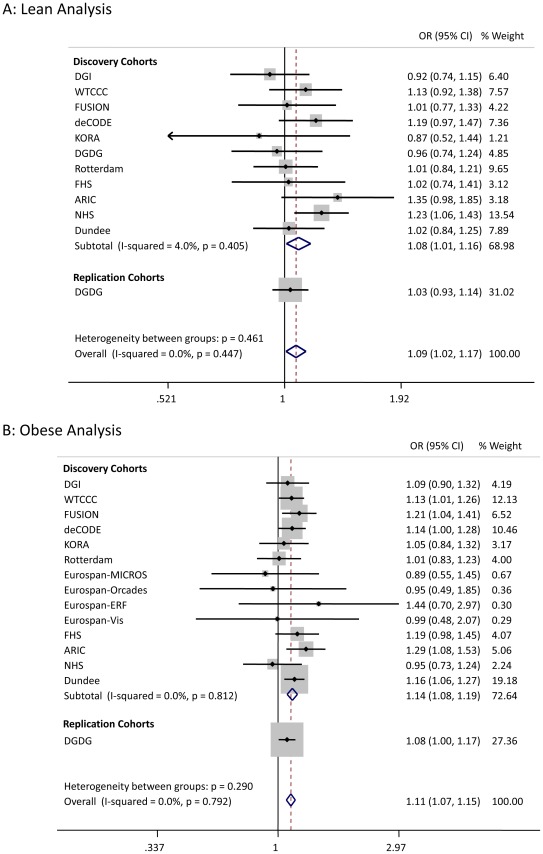
Test statistics for *HMG20A* association in lean and obese cases versus all controls.

**Figure 4:**
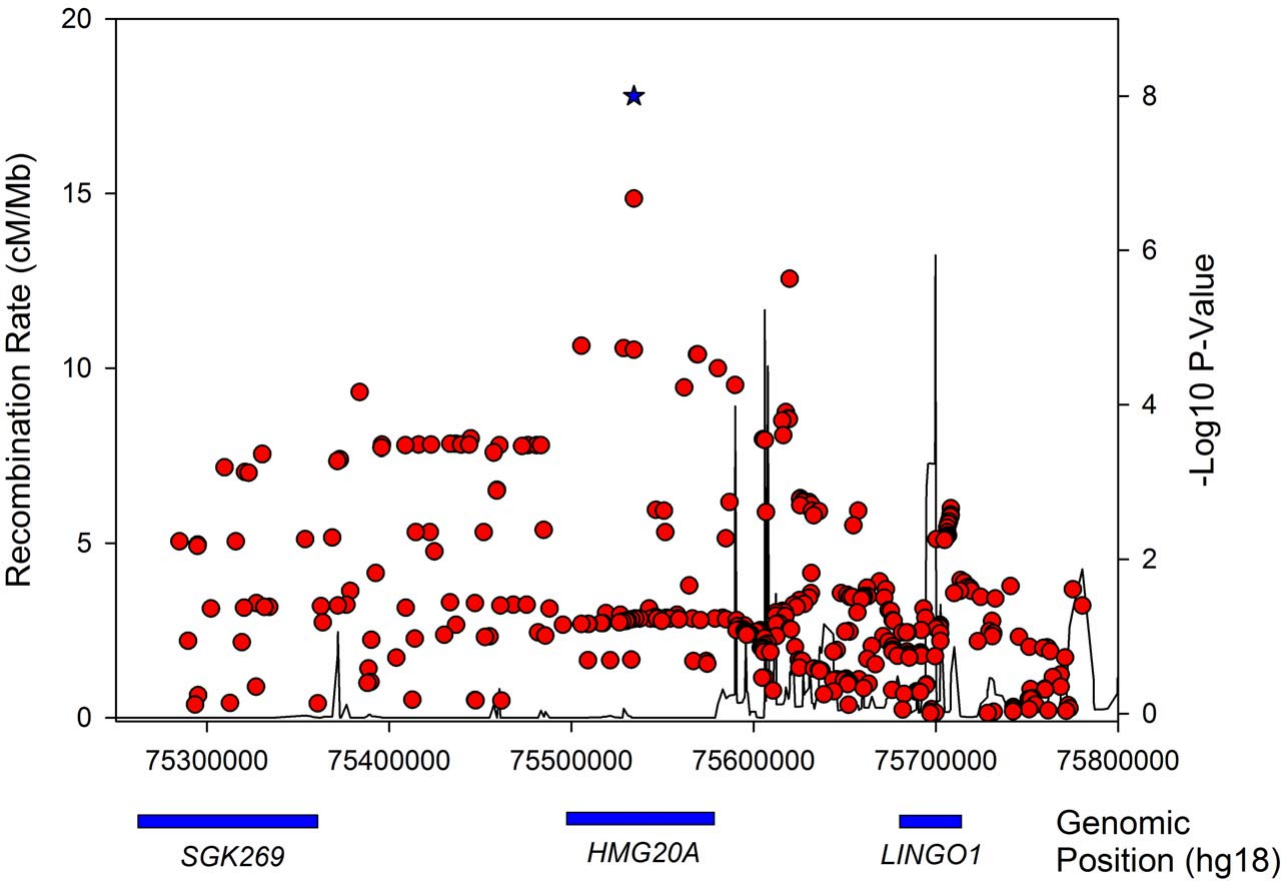
Regional association plot for the *HMG20A* gene in obese type 2 diabetes samples.

We sought to replicate the signals reaching *P*<5×10^−7^ not previously reported in Europeans. SNPs representing the *LAMA1* (rs8090011), *HLA-DQA2* (rs3916765), *HMG20A* (rs7178572), and *ANKS1A* (rs16896390) signals were genotyped in up to 2,881 lean cases, 8,702 obese cases and 18,957 control individuals. Combined discovery and follow-up association statistics for these SNPs are shown in Table 2. In the lean case analysis, the *LAMA1* variant was associated with type 2 diabetes (combined *P* = 8.4×10^−9^, OR = 1.13 [1.09–1.18], total lean cases N = 4,993, controls = 70,515) compared to an OR = 1.03 [1.00–1.06] in the obese case analysis (Figure 1 and Figure 2). In the obese case analysis, the *HMG20A* signal was associated with type 2 diabetes (combined *P* = 1.3×10^−8^, OR = 1.11 [1.07–1.15], total obese cases N = 8,583, controls = 62,063) compared to an OR = 1.09 [1.02–1.17], *P* = 0.015, in the lean analysis (Figure 3 and Figure 4). In previously published studies including 8,130 cases not stratified by BMI [1], the *LAMA1* and *HMG20A* variants reached only nominal levels of significance of *P* = 0.002 (OR = 1.07 [1.03–1.12]) and *P* = 0.003, OR = 1.07 [1.02–1.12] respectively (both in the same directions as reported here).

Considering a random-effects model [39] for both *LAMA1* and *HMG20A* signals gave similar evidence for association (*LAMA1* lean analysis: *P* = 5×10^−10^, obese analysis: *P* = 0.02; *HMG20A* lean analysis: *P* = 0.04, obese analysis: *P* = 2.7×10^−8^). Evidence for association at the *HLA-DQA2* and *ANKS1A* signals was reduced when follow-up data were included.

### Association of variants with BMI

We next attempted to understand further the associations between SNPs in the *LAMA1* and *HMG20A* loci and lean and obese type 2 diabetes cases respectively. Our study design, together with the associations between the *FTO* and *MC4R* variants in the obese strata, suggested that variants that primarily operate through BMI could drive our newly identified associations. We therefore assessed the two signals in the existing GWA studies of BMI performed by the GIANT study and consisting of 123,865 individuals [18]. The *LAMA1* SNP was not associated with BMI (*P* = 0.19) whilst the type 2 diabetes risk allele at the *HMG20A* SNP was nominally associated with increased BMI (*P* = 0.02).

### Association of variants with BMI within cases only

If the associations at the *LAMA1* and *HMG20A* loci are genuinely stronger in one strata of diabetic cases compared to the other, we should observe an association of those variants with BMI within cases only. This phenomenon has previously been reported for the variants in *TCF7L2[14]*. The *LAMA1* type 2 diabetes risk allele was associated with lower BMI within cases alone (*P* = 2×10^−6^ when analysing BMI as a quantitative trait in 26,366 cases), a result consistent with its association being stronger in the lean case analysis. The *HMG20A* risk allele showed no evidence of association (*P*>0.05).

### Association of variants with continuous glycaemic measures

Next we used data from MAGIC to assess potential roles of variants in normal glycaemia. The SNP representing the novel *LAMA1* association showed no association with fasting glucose (*P* = 0.48, beta(se) = 0.0027(0.004) N = 46,186), fasting insulin (*P* = 0.87, beta(se) = 0.0006(0.004) N = 38,238), HbA_1C_ (*P* = 0.19, beta(se) = 0.005(0.004) N = 46,368), 2-hour glucose response (*P* = 0.43, beta(se) = −0.016(0.02), N = 15,234), or any of the SNP×BMI-interaction models. However, *LAMA1* isn't unique amongst type 2 diabetes loci in showing no effect on glycemic traits in the MAGIC study.

The *HMG20A* diabetes risk allele was associated with higher fasting glucose (*P* = 0.04, beta(se) = 0.008(0.004), N = 46,186), higher HbA1C (*P* = 0.002, beta(se) = 0.01(0.004), N = 46,368) and higher fasting glucose after accounting for BMI and SNPxBMI interaction (*P* = 0.008, N = 58,074).

### Association of variants with *cis* gene expression levels

In an attempt to gain further insight into likely functional genes in the *LAMA1* and *HMG20A* loci, we tested the lead SNPs at for association in a number of eQTL datasets. Tissues tested included various blood, brain, liver and fat samples (see Methods). Only ‘*cis*’ associations were considered (eQTL effects on a transcript within 1 Mb of the signal SNPs). The rs7178572 SNP in the *HMG20A* region was significantly associated with mRNA expression levels of *HMG20A* in the liver (*P* = 4×10^−5^), supported by two separate expression probes, and was the strongest known regional SNP for both the liver eQTL and type 2 diabetes. No other study-wide significant results were observed (N = 14 tissues, 24 datasets/analyses).

### Evidence that genetic variants associated with type 2 diabetes have different effects between lean and obese cases

**Figure 5:**
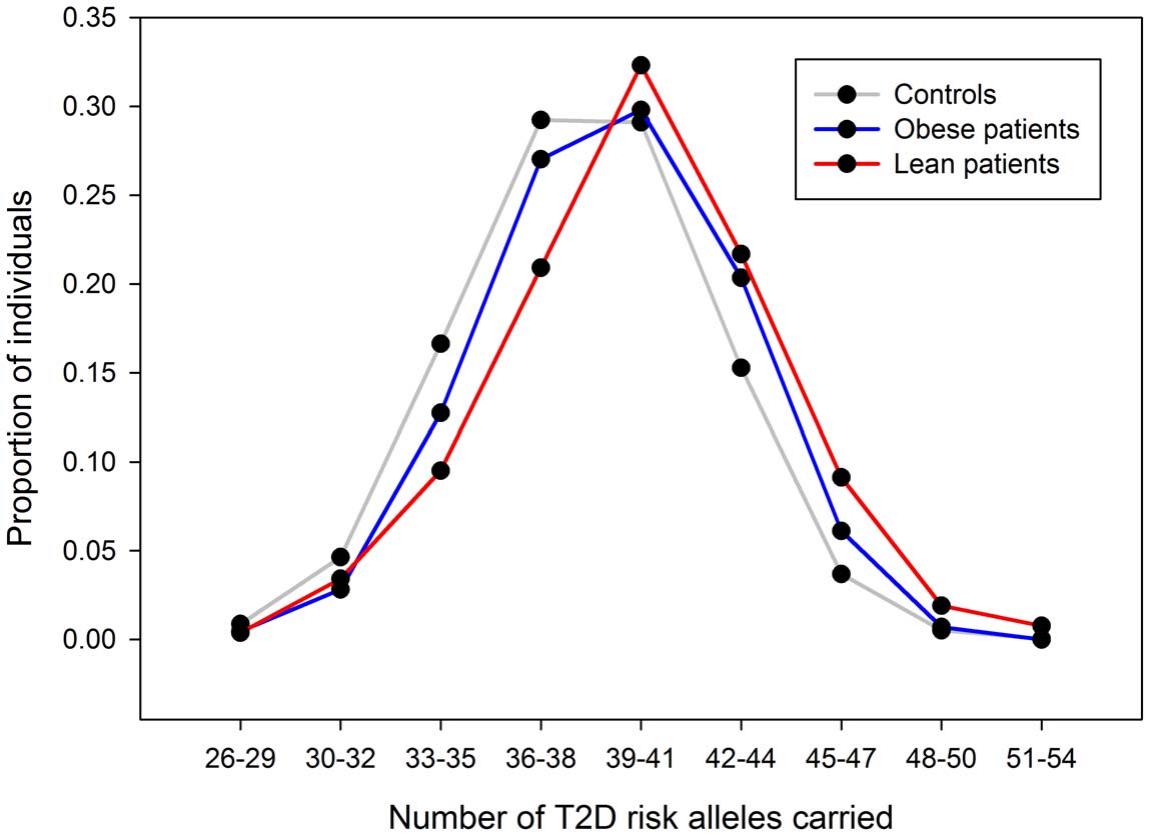
Risk allele distribution for known type 2 diabetes SNPs in GoDARTs. Plot shows number of type 2 diabetes risk alleles carried by the 263 lean type 2 diabetes cases, 1,735 obese type 2 diabetes cases and 3,691 controls from the GoDARTs study.

For each of 36 published type 2 diabetes loci (identified in European studies and available on the metabochip) we compared the effect sizes between the lean and obese GWA study meta-analyses (Table 3). Among the 36 independent variants, 29 had a larger point estimate odds ratio in the lean analysis compared to the obese analysis (binomial test of 29/36 versus 50% under the null hypothesis of no difference, *P* = 0.0002). We next assessed the combined effect of these SNPs in a case control study independent of the GWA studies - GoDARTs (Figure 5). In the lean stratum, we observed a weighted per-risk allele OR = 1.13 [1.10–1.17], *P* = 3.2×10^−14^. This was larger than the same model fitted in the obese strata where the OR = 1.06 [1.05–1.08], *P* = 2.2×10^−16^. Results were very similar when stratifying the controls as well as the cases by BMI: lean weighted per risk-allele OR = 1.13 [1.09–1.17]; obese weighted per risk-allele OR = 1.08 [1.05–1.10] (heterogeneity of odds ratios *P* = 0.036). We also observed a difference between lean and obese cases when removing controls and fitting a regression model of lean cases vs obese cases (P = 0.0001). None of these 36 variants were associated with BMI in 28,000–32,000 individuals from GIANT [1], [2].

**Table 3:** Association statistics for known European type 2 diabetes loci in the lean and obese GWA studies strata.

				Lean T2D Cases N = 2112 vs all controls		Obese T2D Cases N = 4123 vs all controls	
SNP	Nearest or Candidate Gene	Chr	Risk Allele	OR	P-value	OR	*P* value
rs7903146	*TCF7L2*	10	T	1.58[1.47–1.68]	2.0E−40	1.26[1.20–1.32]	4.40E−21
rs11708067	*ADCY5*	3	A	1.25[1.15–1.35]	5.7E−08	1.07[1.01–1.14]	0.01
rs10923931	*NOTCH2*	1	T	1.22[1.11–1.35]	0.00007	1.06[0.98–1.13]	0.14
rs13266634	*SLC30A8*	8	C	1.24[1.15–1.34]	5.1E−08	1.12[1.06–1.19]	0.00005
rs2237892	*KCNQ1*	11	C	1.29[1.12–1.48]	0.0003	1.11[1.01–1.22]	0.02
rs10010131	*WFS1*	4	G	1.14[1.07–1.22]	0.00005	1.07[1.02–1.12]	0.004
rs10811661	*CDKNA/2B*	9	T	1.22[1.12–1.33]	0.000007	1.12[1.05–1.19]	0.0002
rs5215	*KCNJ11*	11	C	1.15[1.08–1.23]	0.00002	1.08[1.03–1.13]	0.0007
rs4457053	*ZBED3*	5	G	1.14[1.05–1.24]	0.002	1.06[1.00–1.12]	0.04
rs340874	*PROX1*	1	C	1.12[1.05–1.19]	0.0008	1.06[1.01–1.11]	0.02
rs7957197	*HNF1A*	12	T	1.07[0.99–1.16]	0.07	1.01[0.96–1.07]	0.69
rs243021	*BCL11A*	2	A	1.12[1.05–1.20]	0.0006	1.07[1.01–1.12]	0.01
rs896854	*TP53INP1*	8	T	1.02[0.96–1.09]	0.44	1.07[1.03–1.12]	0.002
rs757210	*HNF1B*	17	T	1.17[1.05–1.29]	0.003	1.09[1.02–1.17]	0.02
rs7578597	*THADA*	2	T	1.20[1.08–1.33]	0.0008	1.12[1.04–1.20]	0.004
rs1111875	*HHEX*	10	C	1.18[1.10–1.25]	5.3E−07	1.13[1.08–1.18]	6.4E−08
rs972283	*KLF14*	7	G	1.13[1.06–1.20]	0.0002	1.09[1.04–1.14]	0.0003
rs7593730	*RBMS1/ITGB6*	2	C	1.12[1.03–1.22]	0.005	1.07[1.00–1.14]	0.03
rs7754840	*CDKAL1*	6	C	1.19[1.11–1.27]	3.80E−07	1.14[1.09–1.20]	2.0E−08
rs4607103	*ADAMTS9*	3	C	1.03[0.95–1.11]	0.47	1.07[1.01–1.13]	0.01
rs1531343	*HMGA2*	12	C	1.13[1.01–1.27]	0.03	1.20[1.10–1.31]	0.00003
rs7961581	*TSPAN8,LGR5*	12	C	1.17[1.08–1.26]	0.00007	1.13[1.06–1.20]	0.00007
rs8042680	*PRC1*	15	A	1.07[1.00–1.14]	0.05	1.04[0.99–1.09]	0.10
rs11634397	*ZFAND6*	15	G	1.08[1.01–1.16]	0.03	1.05[1.00–1.10]	0.04
rs7578326	*IRS1*	2	A	1.10[1.03–1.18]	0.008	1.07[1.02–1.13]	0.006
rs1552224	*CENTD2*	11	A	1.05[0.96–1.14]	0.27	1.08[1.02–1.15]	0.01
rs1801282	*PPARg*	3	C	1.14[1.04–1.26]	0.008	1.11[1.04–1.18]	0.003
rs12779790	*CAMK1D*	10	G	1.12[1.03–1.22]	0.009	1.09[1.02–1.16]	0.008
rs864745	*JAZF1*	7	T	1.09[1.03–1.16]	0.006	1.08[1.03–1.12]	0.001
rs780094	*GCKR*	2	C	1.04[0.98–1.11]	0.19	1.03[0.98–1.08]	0.22
rs13292136	*TLE4*	9	C	1.15[1.00–1.31]	0.04	1.12[1.00–1.24]	0.04
rs231362	*KCNQ1*	11	G	1.09[1.02–1.17]	0.01	1.08[1.03–1.14]	0.003
rs4607517	*GCK*	7	A	1.03[0.94–1.12]	0.55	1.04[0.98–1.10]	0.20
rs1470579	*IGF2BP2*	3	C	1.15[1.07–1.23]	0.00009	1.16[1.10–1.23]	6.4E−08
rs10830963	*MTNR1B*	11	G	1.11[1.03–1.20]	0.007	1.10[1.04–1.16]	0.0005
rs2191349	*DGKB/TME195*	7	T	1.03[0.97–1.10]	0.38	1.04[0.99–1.08]	0.13

KCNQ1 appears twice as it has two independent signals confirmed through conditional analysis.

**Figure 6:**
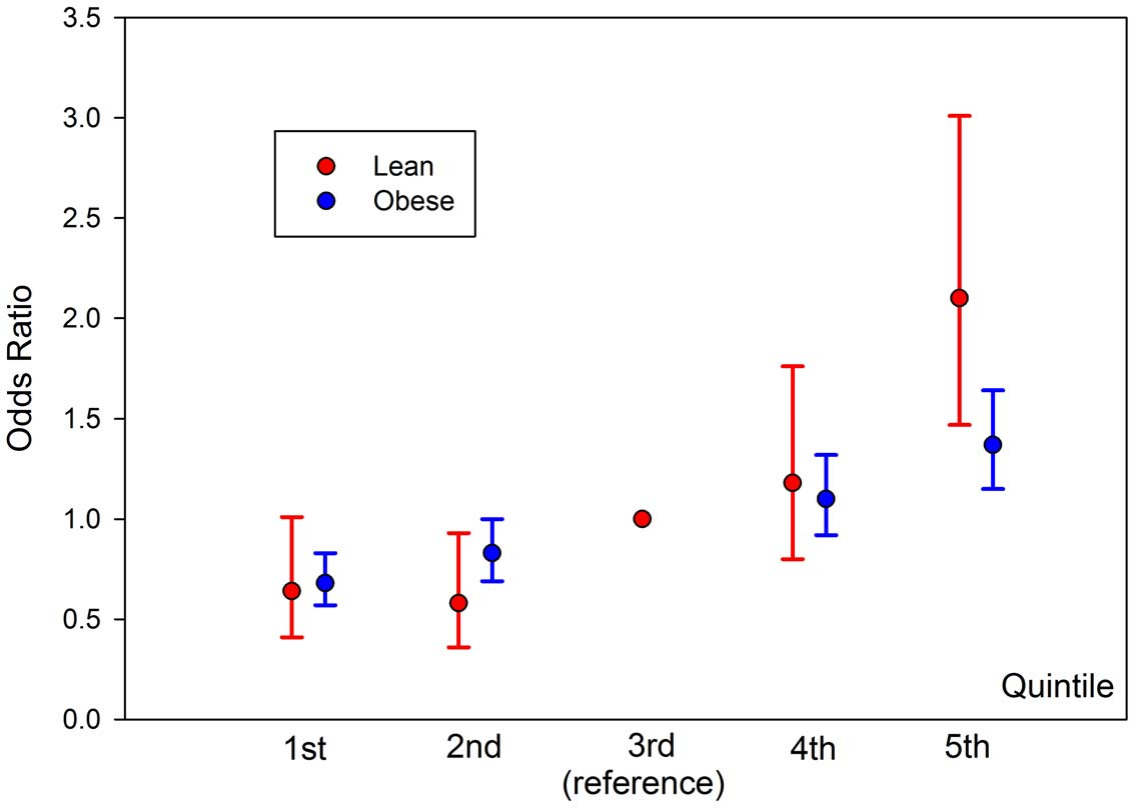
Relative risk for type 2 diabetes depending on risk allele quintile, split by lean and obese BMI. Individuals binned into quintiles based on risk-allele count of known SNPs, weighted by effect size of SNP. Risk estimates relative to median quintile. Total sample size across all quintiles is 263 lean type 2 diabetes cases, 1735 obese type 2 diabetes cases and 3691 controls from the GoDARTs study.

We next divided the case/control samples into risk quintiles, based on the number of risk alleles they carry, weighted by the relative effect sizes of those alleles from the larger DIAGRAM meta-analysis. The risk of being in each quintile relative to the median quintile is shown in Figure 6. For the lean group, we observed an OR = 2.1 [1.47–3.01] for the quintile of individuals carrying the most risk alleles compared to the middle quintile. This effect was larger than that in the obese group where the equivalent OR = 1.37 [1.15–1.64].

## Discussion

We have confirmed our hypothesis that it is possible to identify genetic associations in previously tested samples by constraining the phenotypic heterogeneity of disease cases. By stratifying type 2 diabetes into two well accepted definitions of lean and obese cases, we identified and replicated one locus in each BMI stratum, each previously unreported in European studies: a signal in the *LAMA1* gene in the lean stratum and a signal in the *HMG20A* gene in the obese stratum. Lack of evidence for association with BMI for these two signals in 123,000 individuals [18] argues that these associations are not driven by a primary association with BMI.

There are two reasons why previously undetected genetic associations may be observed in stratified data. First chance, in this context “sampling error”, may occur – new signals may reach statistical thresholds in subsets of data due to a combination of real association and chance. Second, the signal may represent genuine heterogeneity. The enrichment of the *LAMA1* signal in lean type 2 diabetes cases compared to obese cases is likely to be a real effect but the enrichment of the *HMG20A* signal in obese cases is more likely to be due to chance. Whilst we observed some regression to the mean (or “winner's curse”) for the *LAMA1* signal, the effects remained different in lean compared to obese cases in the replication samples alone (Figure 1). In addition, the *LAMA1* type 2 diabetes risk allele was associated with lower BMI within cases alone (*P* = 2×10^−6^ when testing BMI as a quantitative trait in cases) – a similar result was previously reported for the *TCF7L2* risk allele [16]. In contrast there is no evidence that the *HMG20A* signal is stronger in obese replication strata compared to lean replication strata (Figure 3) and there was no association with increased BMI within cases alone (*P*>0.05 when testing BMI as a quantitative trait in cases).

The *LAMA1* signal falls in a recombination block within the *LAMA1* gene (Figure 2), with the lead SNP positioned within intron 61. Searching for correlated SNPs (r^2^>0.5) using 1000 Genomes Project data identified only additional intronic SNPs. Previous cell biology studies support a role for *LAMA1*, encoding laminin-1, in diabetes etiology - inhibition of *LAMA1* expression reduced glucose-stimulated secretion in INS1E cells [40]. Several studies observed the beneficial effects of laminin-1, and extracellular matrix (highly enriched with laminin-1) preparations on pancreatic islet development and function [41], [42], [43], [44], [45], [46]. Laminin-1 is expressed in intra-islet capillaries [47] and a role for laminin receptor 1 was proposed in angiogenesis [48].

The confidence in the *HMG20A* association is enhanced by several lines of evidence from other studies. The *HMG20A* signal was previously identified in a GWA study of South Asian individuals [7] and was nominally associated with fasting glucose (*P* = 0.04, N = 46,186) and HbA1C (*P* = 0.002, N = 46,368) in non-diabetic individuals analysed by the MAGIC consortium. The association with fasting glucose became stronger when adjusting for BMI in an interaction model (*P* = 0.008).

We initially discovered a genome-wide significant signal near the *HLA-DQA2* locus, which subsequently failed to replicate (rs3916765, *P* = 1×10^−6^). This variant is not in the same gene or in linkage disequilibrium with previously reported associations between *HLA* loci and type 2 diabetes [1], [49]. Concerned with the prospect of this association being due to auto-immune diabetes case admixture, we assessed the association of the strongest known type 1 diabetes signals in our lean meta-analysis. None of these showed any significant evidence of association – including the lead signals from the WTCCC type 1 diabetes study in the HLA region (rs3129941, *P* = 0.08), or near the *INS* (rs3842748, *P* = 0.64) or *PTPN22* (rs2476601, *P* = 0.38) genes.

This study has provided the most robust evidence to date that lean type 2 diabetic cases are likely to carry a disproportionately high load of known type 2 diabetes risk alleles. More than 80% (29/36) of type 2 diabetes variants established in Europeans had stronger effects in lean compared to obese cases and the odds ratio for the 20% of lean cases carrying the most risk alleles was more than twice that of the 20% of obese cases carrying the most risk alleles. The corollary of these findings is that obese cases on average carry a disproportionately low load of confirmed type 2 diabetes risk variants, but their diabetes risk will likely be more heavily influenced by their genetic and environmental predisposition to gaining weight in adulthood.

Despite this enrichment of stronger effects in lean versus obese cases, analyses focused only on lean cases is not a more powerful study design compared to using all cases. For each of the known loci tested, the power gained by increased effect sizes is easily offset by the reduced power of having a case sample size of ∼25%. Nevertheless our data indicate that, given limited resources, recruitment strategies that target leaner type 2 diabetes cases will have more power than those that target a similar number of cases but without enrichment for lower BMI.

There are several limitations to our study. First, the use of an unstratified control group made testing the significance of differences between lean and obese cases difficult in the context of a genome wide meta-analysis. However, several lines of evidence support our conclusions that lean individuals are enriched for known type 2 diabetes genetic effects. This evidence includes: the very large differences between the upper and lower 95% confidence intervals of the weighted per allele effects in lean and obese, the consistency of the weighted per allele results when stratifying controls as well as cases, and the 80/20 proportion of SNPs showing stronger effects in lean compared to obese individuals respectively. Second, after stratifying by BMI, we did not use other criteria to reduce the clinical heterogeneity of type 2 diabetes. Of note, cases within the BMI strata differed appreciably in their age at diagnosis and the degree to which autoimmune or monogenic diabetes had been excluded. Instead, having stratified by BMI, we opted to use the largest available sample sizes. It is possible that a small number of monogenic or autoimmune forms of diabetes amongst our cases could have reduced our power to detect novel variants. Further studies may help refine how known and novel diabetes signals operate in more clinically homogenous settings. Finally, known type 2 diabetes signals are likely to account for only a small fraction of all risk variants that exist in the genome and any inferences we make are limited to the known signals.

In conclusion, we report associations with the *LAMA1* and *HMG20A* (not previously associated at genome-wide significance in Europeans) gene regions with type 2 diabetes risk. We have demonstrated that lean diabetic cases are enriched for known type 2 diabetes risk alleles compared to obese cases. This enrichment is consistent with the observation that many of the variants with the strongest effects on diabetes are associated with reduced beta cell function [1]. At the opposite end of the spectrum, obese cases presumably need fewer diabetes risk variants to push them towards diabetes, as they are already under strain from the physiological impact of obesity and insulin resistance. These data suggest a disease model where type 2 diabetes cases lie across a continuous distribution with regards to genetic/environmental risk, and beta-cell dysfunction versus insulin resistance aetiologies.

## Supporting Information

Table S1 Summary characteristics of discovery GWA studies cohorts. Eurospan represents a single cohort in the main text, however is split into its component studies in this table. n/a = not applicable.
(DOC)

Table S2 Summary characteristics of replication cohorts.
(DOC)

Text S1 Full study acknowledgements.
(DOC)
