# Supplementary material for: Stratifying Type 2 Diabetes Cases by BMI Identifies Genetic Risk Variants in LAMA1 and Enrichment for Risk Variants in Lean Compared to Obese Cases
Source: PLoS Genet. 2012 May 31;8(5):e1002741. doi: 10.1371/journal.pgen.1002741 (PMC3364960; doi:10.1371/journal.pgen.1002741)
Supplement: Table S1 — Summary characteristics of discovery GWA studies cohorts. Eurospan represents a single cohort in the main text, however is split into its component studies in this table. n/a = not applicable. (DOC) [file pgen.1002741.s001.doc]

**Supplementary Table 1**

| **Study** | **Case strata** | **N** | **BMI Range** | **BMI Mean (SD)** | **M/F**  **Number** | **% insulin**  **treatment** | **Age of Diagnosis**  **mean(sd)** |
| --- | --- | --- | --- | --- | --- | --- | --- |
|
| ARIC | Lean (BMI <25) | 111 | 19.28-24.98 | 23.06(1.50) | 52/59 | 11.71 | 47.90(12.00) |
| deCODE | Lean (BMI <25) | 214 | 14.7-25 | 23.20 (1.78) | 117/97 | 11.68 | 54.6(14.9) |
| DGDG | Lean (BMI <25) | 185 | 15.11-25 | 22.72(1.81) | 99/86 | 26.46 | 44(9) |
| DGI | Lean (BMI <25) | 225 | 18-25 | 22.93 (1.51) | 106/119 | 12.89 | 59.47(10.57) |
| Eurospan-ERF | Lean (BMI <25) | n/a | n/a | n/a | n/a |  |  |
| Eurospan-Micros | Lean (BMI <25) | n/a | n/a | n/a | n/a |  |  |
| Eurospan-Orcades | Lean (BMI <25) | n/a | n/a | n/a | n/a |  |  |
| Eurospan-Vis | Lean (BMI <25) | n/a | n/a | n/a | n/a |  |  |
| FHS | Lean (BMI <25) | 93 | 18.71-24.97 | 23.07 (1.51) | 47/46 |  | 58(median) |
| FUSION | Lean (BMI <25) | 123 | 16-24.99 | 23.22 (1.61) | 78/45 | 42.27 | 53.66(9.73) |
| KORA | Lean (BMI <25) | 36 | 20.33-24.87 | 23.47 (1.33) | 21/15 | 16.67 | 57.48(11.92) (n=23) |
| NHS | Lean (BMI <25) | 567 | 17.38 - 24.99 | 22.67 (1.67) | 0/100 |  | 57.68(13.37) |
| Rotterdam | Lean (BMI <25) | 301 | 16.65-25 | 22.87(1.58) | 144/157 |  | n/a |
| WTCCC | Lean (BMI <25) | 257 | 17.9-24.9 | 23 (1.54) | 160/97 | 19 | n/a |
| ARIC | Obese(BMI > 30) | 358 | 30.02-53.98 | 34.95(4.27) | 174/184 | 11.73 | 51.40(9.35) |
| deCODE | Obese(BMI > 30) | 625 | 30-72.1 | 34.69 (4.38) | 346/279 | 8.32 | 54.1(11.2) |
| DGDG | Obese(BMI > 30) | n/a | n/a | n/a | n/a | n/a | n/a |
| DGI | Obese(BMI > 30) | 303 | 30-43.77 | 33.1 (2.52) | 143/160 | 10.56 | 56.49(9.90) |
| Eurospan-ERF | Obese(BMI > 30) | 25 | 30.27-43.25 | 34.98 ( 3.80 ) | 14/11 |  |  |
| Eurospan-Micros | Obese(BMI > 30) | 22 | 30.11-45.84 | 34.30 ( 3.65) | 15/7 |  |  |
| Eurospan-Orcades | Obese(BMI > 30) | 21 | 30.93-47.49 | 35.25 ( 4.52 ) | 14/7 |  |  |
| Eurospan-Vis | Obese(BMI > 30) | 38 | 30.09-40.69 | 32.83 ( 2.74 ) | 25/13 |  |  |
| FHS | Obese(BMI > 30) | 331 | 30.00-58.29 | 36.27 (5.33) | 181/150 |  | 56(median) |
| FUSION | Obese(BMI > 30) | 529 | 30-47.59 | 34.0 (3.37) | 265/264 | 38.16 | 53.62(8.72) |
| KORA | Obese(BMI > 30) | 219 | 30.11-51.95 | 34.64 (4.07) | 115/104 | 21 | 56.77(9.82) (n=169) |
| NHS | Obese(BMI > 30) | 394 | 30.04-56.11 | 33.88 (3.77) | 0/100 |  | 56.01(10.10) |
| Rotterdam | Obese(BMI > 30) | 247 | 30.01-49.83 | 33.05(3.02) | 62/185 |  | n/a |
| WTCCC | Obese(BMI > 30) | 1,011 | 30-62.4 | 35.63 (4.98) | 533/478 | 26 | n/a |
| ARIC | Controls | 7,159 | 14.38-56.26 | 26.37(4.50) | 3167/3992 | n/a |  |
| deCODE | Controls | 2,3193 | 13.67-73.5 | 26.78 (5.04) | 7314/15879 | n/a | n/a |
| DGDG | Controls | 670 | 20-27 | 23.20(1.78) | 268/402 | n/a | n/a |
| DGI | Controls | 1,075 | 17.3-43.9 | 27.11 (3.79) | 540/535 | n/a | n/a |
| Eurospan-ERF | Controls | 1,178 | 15.54-51.13 | 26.57 (4.6) | 463/715 | n/a | n/a |
| Eurospan-Micros | Controls | 1,018 | 14.03-48.91 | 25.36 (4.45) | 437/581 | n/a | n/a |
| Eurospan-Orcades | Controls | 668 | 16.97-47.63 | 27.54 (4.3) | 306/362 | n/a | n/a |
| Eurospan-Vis | Controls | 660 | 17.01-52.02 | 27.15 (4.23) | 276/384 | n/a | n/a |
| FHS | Controls | 7,570 | 13.81-64.07 | 27.03 (5.14) | 3418/4152 | n/a | n/a |
| FUSION | Controls | 1,174 | 17.50-51.06 | 27.14 (3.86) | 574/600 | n/a | n/a |
| KORA | Controls | 1,432 | 16.77-51.43 | 27.72 (4.26) | 691/741 | n/a | n/a |
| NHS | Controls | 1,754 | n/a | n/a | n/a | n/a | n/a |
| Rotterdam | Controls | 4,593 | 14.18-50.69 | 26.03(3.55) | 1928/2833 | n/a | n/a |
| WTCCC | Controls | 2,938 | 13.4-53.2  (N=1422) | 26.2 (4.34) | 687/735 | n/a | n/a |
