# Supplementary material for: Stratifying Type 2 Diabetes Cases by BMI Identifies Genetic Risk Variants in LAMA1 and Enrichment for Risk Variants in Lean Compared to Obese Cases
Source: PLoS Genet. 2012 May 31;8(5):e1002741. doi: 10.1371/journal.pgen.1002741 (PMC3364960; doi:10.1371/journal.pgen.1002741)
Supplement: Table S2 — Summary characteristics of replication cohorts. (DOC) [file pgen.1002741.s002.doc]

**Supplementary Table 2**

| **Study** | **Strata** | **N** | **BMI Range** | **BMI Mean**  **(SD)** | **M/F ratio** | **% insulin**  **treatment** | **%**  **OHAs** | **% diet**  **controlled** | **Age of Diagnosis**  **mean(sd)** |
| --- | --- | --- | --- | --- | --- | --- | --- | --- | --- |
|
| goDARTS | Lean (BMI <25) | 263 | 16.2-24.9 | 22.97 (1.77) | 151/112 | 27 | 53 | 20 | 56.83 (8.95) |
| DGDG | Lean (BMI <25) | 1161 | 16-24.98 | 22.53 (1.51) | 680/530 | 24.38 | 45.31 | NA | 48.00 (10.00) |
| Malmo | Lean (BMI <25) | 477 | 14.3-25 | 22.9 (1.9) | 291/186 | 20 | NA | 29 | 59.2 (11.6) |
| ADDITION-Ely | Lean (BMI <25) | 39 | 15.98-24.95 | 23.31(1.57) | 27/12 | NA | NA | NA | 66.59 (5.20) |
| NDCCS | Lean (BMI <25) | 941 | 15.68-24.99 | 22.96(1.76) | 544/397 | NA | NA | NA | 63.35 (12.29) |
| goDARTS | Obese (BMI > 30) | 1735 | 30-62.3 | 35.94 (5.33) | 950/785 | 19 | 60 | 21 | 54.8 (8.96) |
| DGDG | Obese (BMI > 30) | 3103 | 30-68.83 | 34.43 (4.36) | 1599/1504 | 33.64 | 41.22 | NA | 49.00 (11.00) |
| Malmo | Obese (BMI > 30) | 1080 | 30-60.3 | 34.7 (4.3) | 583/497 | 17 | NA | 0.31 | 55.8 (10.8) |
| ADDITION-Ely | Obese (BMI > 30) | 586 | 30.0-55.13 | 35.69(4.78) | 346/240 | NA | NA | NA | 60.67 (7.65) |
| NDCCS | Obese (BMI > 30) | 2329 | 30.0-72.24 | 35.04(4.81) | 1208/1121 | NA | NA | NA | 58.01(11.25) |
| goDARTS | Controls | 3691 | 14.3-54.9 | 27.04 (4.52) | 1913/1778 | NA | NA | NA | NA |
| DGDG | Controls | 4077 | 16-48 | 25.15 (4.02) | 1767/2310 | NA | NA | NA | NA |
| Malmo | Controls | 3470 | 15.6-43 | 25(3.6) | 1310/2160 | NA | NA | NA | NA |
| ADDITION-Ely | Controls | 1518 | 16.89-59.28 | 27.18 (4.74) | 694/824 | NA | NA | NA | NA |
| NDCCS | Controls | 6318 | 15.71-72.32 | 26.08 (3.61) | 2992/3326 | NA | NA | NA | NA |
