# Supplementary material for: Stratifying Type 2 Diabetes Cases by BMI Identifies Genetic Risk Variants in LAMA1 and Enrichment for Risk Variants in Lean Compared to Obese Cases
Source: PLoS Genet. 2012 May 31;8(5):e1002741. doi: 10.1371/journal.pgen.1002741 (PMC3364960; doi:10.1371/journal.pgen.1002741)
Supplement: Text S1 — Full study acknowledgements. (DOC) [file pgen.1002741.s003.doc]

**Full Study Acknowledgements**

J.R.B.P is supported by the Wellcome Trust as a Sir Henry Wellcome Postdoctoral Research Fellow (092447/Z/10/Z). This work was partially funded by grants from the Wellcome Trust 083270/Z/07/Z and MRC G0601261. The Atherosclerosis Risk in Communities Study is carried out as a collaborative study supported by National Heart, Lung, and Blood Institute contracts (HHSN268201100005C, HHSN268201100006C, HHSN268201100007C, HHSN268201100008C, HHSN268201100009C, HHSN268201100010C, HHSN268201100011C, and HHSN268201100012C), R01HL087641, R01HL59367 and R01HL086694; National Human Genome Research Institute contract U01HG004402; National Institutes of Health contract HHSN268200625226C and grants DK062370 and DK072193The authors thank the staff and participants of the ARIC study for their important contributions. Infrastructure was partly supported by Grant Number UL1RR025005, a component of the National Institutes of Health and NIH Roadmap for Medical Research. Work at Lund University diabetes centre was funded by several grants from the Swedish Research Council (LG prject grant, Linné, Exodiab). Norfolk Diabetes Case-Control and ADDITION-Ely Studies: The work on Ely, ADDITION and EPIC-Norfolk studies was funded by support from the Wellcome Trust and MRC. The Norfolk Diabetes study is funded by the MRC with support from NHS Research & Development and the Wellcome Trust and we are grateful to the study team. This research was conducted in part using data and resources from the Framingham Heart Study of the National Heart Lung and Blood Institute of the National Institutes of Health and Boston University School of Medicine. The analyses reflect intellectual input and resource development from the Framingham Heart Study investigators participating in the SNP Health Association Resource (SHARe) project. This work was partially supported by the National Heart, Lung and Blood Institute's Framingham Heart Study (Contract No. N01-HC-25195) and its contract with Affymetrix, Inc for genotyping services (Contract No. N02-HL-6-4278). A portion of this research utilized the Linux Cluster for Genetic Analysis (LinGA-II) funded by the Robert Dawson Evans Endowment of the Department of Medicine at Boston University School of Medicine and Boston Medical Center. Also supported by National Institute for Diabetes and Digestive and Kidney Diseases (NIDDK) R01 DK078616 to Drs. Meigs and Dupuis, and NIDDK K24 DK080140 to Dr. Meigs. The research performed at deCODE Genetics was part funded through the European Community's Seventh Framework Programme (FP7/2007-2013), ENGAGE project, grant agreement HEALTH-F4-2007- 201413. The DGDG study was supported by the French Government (Agence Nationale de la Recherche), the French Region of Nord Pas De Calais (Contrat de Projets État-Région), Programme Hospitalier de Recherche Clinique (French Ministry of Health), and the following charities: Association Française des Diabétiques, Programme National de Recherche sur le Diabète, Association de Langue Française pour l'Etude du Diabète et des Maladies Métaboliques, Association Diabète Risque Vasculaire (Paris, France) and Groupe d'Etude des Maladies Métaboliques et Systémiques. This study was also supported in part by a grant from the European Union (Integrated Project EuroDia LSHM-CT-2006-518153 in the Framework Programme 6 [FP6] of the European Community). The D.E.S.I.R. study was supported by the Caisse Nationale d’Assurance Maladie des Travailleurs Salariés, Lilly, Novartis Pharma and Sanofi-Aventis, Institut National de la Santé et de la Recherche Médicale (INSERM) (Réseaux en Santé Publique, Interactions entre les déterminants de la santé, Cohortes Santé TGIR 2008), Association Diabète Risque Vasculaire, Fédération Française de Cardiologie, Fondation de France, Association de Langue Francaise pour l'Etude du Diabete et des Maladies Metaboliques, Office National Interprofessionnel des Vins, Ardix Medical, Bayer Diagnostics, Becton Dickinson, Cardionics, Merck Santé, Novo Nordisk, Pierre Fabre, Roche and Topcon. The D.E.S.I.R. Study Group: INSERM 1018: B. Balkau, P. Ducimetière, E. Eschwège; INSERM U367: F. Alhenc-Gelas; Centre Hospitalier Universitaire D'Angers: Y. Gallois, A. Girault; Bichat Hospital: F. Fumeron, M. Marre, R. Roussel; CHU de Rennes: F. Bonnet; CNRS UMR8199, Lille: P. Froguel; Medical Examination Services: Alençon, Angers, Blois, Caen, Chartres, Chateauroux, Cholet, Le Mans, Orléans and Tours; Research Institute for General Medicine: J. Cogneau; General practitioners of the region; Cross-Regional Institute for Health: C. Born, E. Caces, M. Cailleau, J.G. Moreau, F. Rakotozafy, J. Tichet, S. Vol. T. We are grateful to all patients for participation in the genetic study. We also thank Marianne Deweirder, Frédéric Allegaert (UMR CNRS 8199, Genomic and Metabolic Disease, Lille, France) for their technical assistance and their precious management of DNA samples. This work was partially funded by grants from the Wellcome Trust 083270/Z/07/Z and MRC G0601261. This work was presented as a poster at the American Diabetes Association's scientific sessions June 2011. The NHS/HPFS T2D GWA study (U01HG004399) is a component of a collaborative project that includes 13 other GWA study funded as part of the Gene Environment-Association Studies (GENEVA) under the NIH Genes, Environment and Health Initiative (GEI) (U01HG004738, U01HG004422, U01HG004402, U01HG004729, U01HG004726, U01HG004735, U01HG004415, U01HG004436, U01HG004423, U01HG004728, RFAHG006033) with additional support from individual NIH (NIDCR:U01DE018993, U01DE018903; NIAAA: U10AA008401, NIDA: P01CA089392,R01DA013423; NCI: CA63464, CA54281, CA136792, Z01CP010200). EUROSPAN cohorts were supported by the European Union framework program 6 EUROSPAN project (contract no. LSHG-CT-2006-018947). The **ERF** study was supported by grants from the NWO, Erasmus MC and the Centre for Medical Systems Biology (CMSB). We are grateful to all patients and their relatives, general practitioners and neurologists for their contributions and to P. Veraart for her help in genealogy, Jeannette Vergeer for the supervision of the laboratory work and P. Snijders for his help in data collection. **MICROS:** The MICROS study is part of the genomic health care program 'GenNova' and was carried out in three villages of the Val Venosta on the populations of Stelvio, Vallelunga and Martello. We thank the primary care practitioners Raffaela Stocker, Stefan Waldner, Toni Pizzecco, Josef Plangger, Ugo Marcadent and the personnel of the Hospital of Silandro  (Department of Laboratory Medicine) for their participation and collaboration in the research project. In South Tyrol, the study was supported by the Ministry of Health and Department of Educational Assistance, University and Research of the Autonomous Province of Bolzano and the South Tyrolean Sparkasse Foundation. The **VIS** study in the Croatian island of Vis was supported through the grants from the Medical Research Council UK and Ministry of Science, Education and Sport of the Republic of Croatia (number 108‐1080315‐0302). The authors collectively thank a large number of individuals for their individual help in organizing, planning and carrying out the field work related to the project and data management: Professor Pavao Rudan and the staff of the Institute for Anthropological Research in Zagreb, Croatia (organization of the field work, anthropometric and physiological measurements, and DNA extraction); Professor Ariana Vorko‐Jovic and the staff and medical students of the Andrija Stampar School of Public Health of the Faculty of Medicine, University of Zagreb, Croatia (questionnaires, genealogial reconstruction and data entry); Dr Branka Salzer from the biochemistry lab “Salzer”, Croatia (measurements of biochemical traits); local general practitioners and nurses (recruitment and communication with the study population); and the employees of several other Croatian institutions who participated in the field work, including but not limited to the University of Rijeka and Split, Croatia; Croatian Institute of Public Health; Institutes of Public Health in Split and Dubrovnik, Croatia. SNP Genotyping of the Vis samples was carried out by the Genetics Core Laboratory at the Wellcome Trust Clinical Research Facility, WGH, Edinburgh. **ORCADES** was supported by the Chief Scientist Office of the Scottish Government, the Royal Society, the MRC Human Genetics Unit and Arthritis Research UK. DNA extractions were performed at the Wellcome Trust Clinical Research Facility in Edinburgh. We would like to acknowledge the invaluable contributions of Lorraine Anderson and the research nurses in Orkney, the administrative team in Edinburgh and the people of Orkney. The research within the **KORA** study was partially funded by the German Center for Diabetes Research (DZD), the Helmholtz Zentrum München, Neuherberg, Germany and supported by grants from the German Federal Ministry of Education and Research the Federal Ministry of Health, the Ministry of Innovation, Science, Research and Technology of the state North Rhine-Westphalia, the German National Genome Research Network (NGFN) and the Munich Center of Health Sciences (MC Health) as part of LMUinnovativ. The research of Inga Prokopenko is funded in part through the European Community's Seventh Framework Programme (FP7/2007-2013),  ENGAGE project, grant agreement HEALTH-F4-2007- 201413.
